# Urethritis durch Koinfektion mit Neisseria gonorrhoeae und Enterobius vermicularis bei einem 27-jährigen Patienten

**DOI:** 10.1007/s00120-022-01958-6

**Published:** 2022-11-04

**Authors:** Eva Schadelbauer, Katharina Tripolt-Droschl, Birgit Sadoghi

**Affiliations:** Abteilung für Dermatologie und Venerologie, Medizinische Universität Graz, Auenbruggerplatz 8, 8036 Graz, Österreich

**Keywords:** Sexuell übertragbare Infektion, Enterobiasis, Gonorrhoe, Oxyuren, Dysurie, Sexually transmitted infection, Enterobiasis, Gonorrhea, Oxyurid, Dysuria

## Abstract

Eine Urethritis wird oft durch sexuell übertragbare Pathogene (beispielsweise *Chlamydia trachomatis *[CT] und *Neisseria gonorrhoeae *[NG]) hervorgerufen [1]. Infektionen mit NG sind eine globale Bürde, da sich jährlich etwa 90 Mio. Erwachsene infizieren [2]. Die Behandlung soll nach Empfehlungen nationaler Richtlinien und Resistenzprofilen erfolgen [2, 3]. *Enterobius vermicularis* (EV) ist der häufigste humane Helminth [4, 5]. Schätzungen zufolge sind weltweit bis zu 1 Mrd. Menschen infiziert [6]. Symptome beinhalten analen Pruritus, wobei viele Infektionen asymptomatisch verlaufen [4–7]. Die Autoren beschreiben den Fall einer Urethritis durch simultane Infektion mit NG und EV.

## Abstract (en)

Urethritis is mainly caused by sexually transmitted pathogens, such as *Chlamydia trachomatis *(CT) and *Neisseria gonorrhoeae *(NG) [1]. Close to 90 million adults are infected annually with NG [2]. Treatment shall be performed according to national guidelines and resistance profiles [2, 3]. *Enterobius vermicularis* (EV) is the most common helminth in humans with 1 billion people globally infected [4–6]. Symptoms include anal pruritus; however, many infections are asymptomatic [4–7]. A case of urethritis due to simultaneous infection with NG and EV is reported.

## Anamnese

Ein 27-jähriger Patient ohne Begleiterkrankungen oder Dauermedikation stellte sich im Mai 2019 wegen seit dem Vortag bestehenden, brennenden Sensationen und Juckreiz im Bereich der Harnröhre sowie transparent-weißlichem Ausfluss vor.

Den letzten ungeschützten Sexualkontakt (welcher genitoanal war) hatte der Patient 7 Tage zuvor mit einer weiblichen Zufallsbekanntschaft.

## Befunde

**Figure 1:**
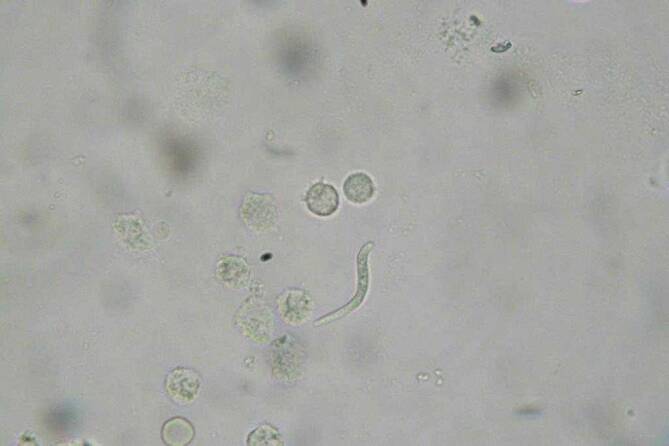

In der körperlichen Untersuchung zeigte sich ein mukoider, transparenter, urethraler Ausfluss. Die regionären Lymphknoten waren inspektorisch als auch palpatorisch insuspekt. In der Gram-Färbung des Urethralsekrets zeigten sich paarig angeordnete, intrazellulär gelegene, Gram-negative Diplokokken sowie eine Akkumulation von Leukozyten. Zusätzlich kamen in der Nativmikroskopie einige weißliche, fadenförmige Formationen zum Vorschein (Abb. 1).

## Therapie und Verlauf

Die Gonorrhö wurde nicht nur mikroskopisch, sondern auch mittels Polymerase Ketten Reaktion(PCR)-Untersuchung bestätigt. Zur leitliniengerechten Therapie der Gonorrhö erhielt der Patient 500 mg Ceftriaxon i.v. kombiniert mit 1,5 g Azithromyzin p.o. als Einmalgaben. Die fadenförmigen Strukturen konnten unter dem Mikroskop als *Enterobius-vermicularis* Wurmlarven identifiziert werden und der Patient erhielt zusätzlich 100 mg Mebendazol p.o. für 3 Tage.

Andere sexuell übertragbare Erkrankungen konnten ausgeschlossen werden. Die Resistenztestung des Gonokokkenisolats zeigte eine Sensibilität auf die verabreichten Antibiotika.

Eine Kontrolluntersuchung wurde aufgrund der EV-Infektion eine Woche nach erfolgter Therapie vereinbart. Im Rahmen der Kontrollvisite konnten im gefärbten Abstrich keine lebenden Wurmlarven mehr nachgewiesen werden. Ein „test of cure“ (TOC) auf Gonorrhö, welcher 3 Wochen nach erfolgter Therapie durchgeführt wurde, zeigte ein negatives Ergebnis. Die Beschwerden des Patienten verschwanden innerhalb weniger Tage nach Therapie.

## Diskussion

Die Urethritis stellt eines der häufigsten urogenitalen Krankheitsbilder dar, weshalb PatientInnen an einer Ambulanz für sexuell übertragbare Erkrankungen vorstellig werden. Die Urethritis wird in den meisten Fällen durch sexuell übertragbare Pathogene wie *Neisseria gonorrhoeae, Chlamydia trachomatis *und* Mycoplasma genitalium* hervorgerufen [1, 8].

Seltenere Pathogene, welche zu Urethritiden führen können, sind beispielsweise *Trichomonas vaginalis, Ureaplasma urealyticum* oder Herpes-simplex-Viren [1].

*Neisseria gonorrhoeae* (NG) ist das obligat humane Bakterium, welches die zweithäufigste sexuell übertragbare Infektion in der Europäischen Union, die Gonorrhö, verursacht [2]. Mit einer Anzahl von insgesamt 100.673 bestätigten Fällen im Jahr 2018 ist die Inzidenz der Gonorrhö in Europa seit 2008 um etwa 240 % angestiegen [2]. NG ist aufgrund seiner Eigenschaft, sich an das Immunsystem seines Wirtes anpassen zu können und auch aufgrund der zunehmenden antimikrobiellen Resistenzen weltweit zu einer therapeutischen Herausforderung geworden [2]. Aus diesem Grund sind Resistenztestungen, eine adäquate Therapie und das Follow-up der betroffenen PatientInnen nicht nur ausschlaggebend für die Krankheitskontrolle, sondern auch um die Ausbreitung von Resistenzen zu vermeiden [2, 3].

Der EV ist einer der häufigsten Parasiten weltweit und weist einen einzigartigen Lebenszyklus auf [5, 7]. Nach oraler oder nasaler Ingestion infektiöser Eier schlüpfen Larven im proximalen Anteil des Dünndarms [5, 7]. Auf ihrem Weg zum Dickdarm häuten sich die Larven 2‑mal, werden zu adulten Würmern und vermehren sich [7].

Der männliche Wurm kann bis zu 50 Tage alt werden und stirbt nach der Kopulation, wohingegen weibliche Exemplare i. Allg. bis zu 100 Tage alt werden [5, 7]. Lediglich der schwangere Wurm, welcher bis zu 11.000 Eier produzieren kann, wandert in die Perianal- und Perinealregion, um dort seine asymmetrischen ovalen Eier zu legen [5].

Der symptomatische Befall mit EV wird auch als Enterobiasis bezeichnet [4, 7]. Das typische klinische Symptom ist nächtlicher, (peri)analer Pruritus, welcher zu unaufhörlichem Kratzen und zu bakterieller Superinfektion führen kann [5–7]. EV kann über Kontakt mit kontaminierten Lebensmitteln, Kleidung, Bettwäsche und anderen Gegenständen bzw. durch direkten Hautkontakt und Sexualkontakt übertragen werden [5, 6, 9].

Zur oralen Therapie stehen effektive Wirkstoffe wie Mebendazol, Pyrantel-Embonat und Pyrvinium-Embonat zur Verfügung [7]. Es ist essenziell, dass alle in einem Haushalt lebenden Personen und auch SexualpartnerInnen der PatientInnen untersucht, getestet und gegebenenfalls ebenfalls behandelt werden [7]. Auch nach erfolgreicher Therapie kann es häufig zu Rezidiven kommen [4, 7].

Der EV wird sehr selten an extraintestinalen Lokalisationen angetroffen und die Diagnose kann in diesen Fällen sehr herausfordernd sein [7, 9].

Mit der Verwendung nativer Mikroskopie vor einer Färbung des Präparats können neben Hefe und Hyphen sowie Myzel auch wesentliche Parasiten wie z. B. *Trichomonas vaginalis* oder auch seltenere Pathogene, wie Oxyuren nachgewiesen werden. Natürlich beruht die Korrektheit des Ergebnisses der nativen Diagnostik auf der Erfahrung der Untersuchenden.

Es gibt vereinzelt Fallberichte, in denen EV an atypischen Lokalisationen wie beispielsweise in der Vagina, der Harnblase, den Nieren, dem Peritoneum, der Leber, den Lungen und den Augäpfeln vorgefunden wurde [5, 7, 9]. Darüber hinaus findet man in der Literatur Fälle von urogenitalen EV-Manifestationen, einerseits bei jungen Mädchen, aber auch bei Männern postkoital [4, 5, 9].

Da der Patient kurze Zeit vor dem Auftreten seiner Beschwerden Analverkehr hatte, erscheint der Sexualkontakt in diesem Fall als wahrscheinlichster Übertragungsweg, durch welchen der Helminth die Urethra des Patienten erreichen konnte. Es ist wahrscheinlich anzunehmen, dass die Dysurie und der Ausfluss durch die NGInfektion hervorgerufen wurden, allerdings kann nicht ausgeschlossen werden, dass die Beschwerden auch Folge der EV-Besiedelung der Urethra waren.

## Fazit

Trotz der seltenen, extraintestinalen Manifestationen von EV kann an eine Enterobiasis gedacht werden, wenn sich PatientInnen mit urethralen Beschwerden präsentieren, insbesondere, wenn diese oral-analen und genitoanalen Geschlechtsverkehr haben. Daher ist die Erhebung einer präzisen und wertfreien Sexualanamnese unerlässlich, da diese, wie auch in diesem Fall, häufig wegweisend für die Diagnose ist. Es sollte auch stets die mikroskopische Analyse des Urethralabstrichs als point-of-care(POC)-Testung angedacht werden.
